# 基于共价有机框架材料的固相微萃取研究进展

**DOI:** 10.3724/SP.J.1123.2024.01002

**Published:** 2025-02-08

**Authors:** Yingchao CHENG, Yiyang GAO, Xiaomin LI, Luyu CHEN, Fang DU, Jie GUO, Yitong MENG, Min SUN, Juanjuan FENG

**Affiliations:** 济南大学化学化工学院,山东济南250022; School of Chemistry and Chemical Engineering, University of Jinan, Jinan 250022, China

**Keywords:** 共价有机框架, 固相微萃取, 色谱分析, 环境分析, 食品分析, 生物分析, 综述, covalent organic frameworks (COFs), solid-phase microextraction (SPME), chromatographic analysis, environmental analysis, food analysis, bioanalysis, review

## Abstract

固相微萃取(SPME)是一种快速、简便的样品前处理技术,能够实现分析物的富集,并便于与其他检测技术联用,从而建立准确、灵敏的分析方法。SPME技术已被广泛应用于环境监测、食品安全、生命分析、生物医药等诸多领域。SPME技术的核心是萃取涂层,萃取涂层的性能直接决定着萃取选择性、萃取效率和富集效应等,因此新型、高性能萃取涂层材料的开发一直是分析化学及样品前处理领域的热点。共价有机框架(COFs)是一种通过共价键连接形成的多孔结晶性网状高分子材料,具有比表面积大、孔隙率高、稳定性好、可设计性强、合成及后修饰简便等优点,已广泛应用于气体吸附、催化、传感和药物输送等领域。近年来,COFs在样品前处理领域引起了广泛关注,多种基于COFs的新型SPME材料被开发出来,并应用于不同类型样品中多种分析物的萃取和富集。本文主要总结了近三年COFs在纤维SPME、管内SPME以及膜SPME等方面的研究进展。通过物理涂覆、原位合成以及化学键合的方法,将功能化COFs或COFs杂化材料修饰于纤维表面,制备出SPME纤维;将SPME纤维与色谱分析技术联用,可用于环境和食品样品中多环芳烃、邻苯二甲酸酯类化合物、多氯联苯类化合物和农药等多种分析物的检测,并能够展现出良好的富集能力、较宽的线性范围和较高的灵敏度。基于管内SPME技术,COFs基整体柱型萃取管和纤维填充型萃取管能够与高效液相色谱在线联用,分别开发出针对合成酚类抗氧化剂和双酚类化合物的高灵敏检测方法。此外,COFs也被应用于膜SPME技术,并能够实现环境水中痕量多氯联苯的高效萃取。最后,本文展望了COFs在SPME领域的发展趋势。

## Abstract (en)

Solid-phase microextraction (SPME) is a fast and simple sample preparation technique that enables the enrichment of analytes, and it is used in combination with other detection techniques to provide accurate and sensitive analytical methods. SPME is widely used in environmental monitoring, food safety, life analysis, biomedicine, and other applications. The extractive coating is the core of the SPME technique, and the properties of the extractive coating greatly influence extraction selectivity and efficiency, as well as the enrichment effect. Therefore, the development of new and efficient extractive coating materials remains a hot topic in the analytical chemistry and sample preparation fields. Covalent organic frameworks (COFs) are a kind of porous crystalline network polymer materials formed by covalent bonds. Owing to the advantages of large specific surface area, high porosity, good stability, high designability, simple synthesis and post-modification, etc., it has been widely used in gas adsorption, catalysis, sensing and drug delivery. In recent years, COFs have attracted much attention in the field of sample preparation. A variety of novel COF-based SPME materials had been developed for extracting and enriching various types of analytes through *π*-*π* interaction, hydrophilic/hydrophobic interaction, electrostatic adsorption, and hydrogen-bonding, as well as pore effects. In this paper, the research advances of COFs for using in fiber-, in tube-, and membrane-based SPME over the past three years were discussed. Fiber surfaces had been modified with functionalized COFs or COF-hybrid materials for use in SPME through physical coating, in-situ growth, and chemical-bonding approaches. The combination of SPME fiber and chromatographic analysis can be used to detect a variety of analytes such as polycyclic aromatic hydrocarbons, phthalates, polychlorinated biphenyls, and pesticides in environmental and food samples, with good enrichment effects, wide linear ranges, and high sensitivity. Based on in tube-SPME, COFs-based monolithic column and fiber-filled tube as the extraction tubes were combined with high performance liquid chromatography online to develop highly sensitive detection methods for synthetic phenolic antioxidants and bisphenol compounds, respectively. In addition, COFs had also been used in membrane SPME technique, showing high efficiency in the extraction of trace polychlorinated biphenyls in environmental water. Finally, the development trends of COFs in the field of SPME was prospected.

样品分析速度和分析结果的准确性会直接受到样品前处理技术的影响。传统的样品前处理方法(如固相萃取、液相萃取等)存在操作步骤繁琐、耗时长、有机溶剂用量大等缺点,难以满足现代分析化学中高效、快速、环保的发展要求,因此开发高效的样品前处理方法至关重要。固相微萃取(SPME)有效地弥补了上述缺陷,它具有操作简便、萃取效率高、萃取速度快、溶剂用量少等优点^[1]^;并且,SPME可以与气相色谱(GC)或高效液相色谱(HPLC)等技术联用,实现快速和高灵敏度的检测。根据萃取材料的不同,SPME可以分为纤维SPME、管内SPME以及膜SPME等多种萃取形式,它们已被广泛应用于各类化合物的分析检测^[2]^。萃取涂层是SPME技术的核心,近年来已有多种先进材料用于萃取涂层的制备,并应用在SPME领域,其中包括聚合离子液体^[3]^、石墨烯^[4]^、纳米材料^[5]^、气凝胶^[6]^、金属有机框架材料^[7]^、共价有机框架(COFs)材料^[8]^等。

COFs的制备是基于拓扑学原理,利用共价键将有机构筑单元进行可逆连接而得到的一种晶态有机多孔材料^[9]^。COFs因具有密度低、比表面积高、结构可调性强、稳定性好和活性位点丰富等优势,受到越来越多的关注。此外,通过功能化或杂化的方法可以进一步提升COFs的性能,目前COFs材料已被广泛应用于各个领域^[10]^。近年来,COFs已被用作SPME材料,广泛应用于环境、食品、生物等领域的样品分析。本文总结了近三年COFs材料在纤维SPME、管内SPME以及膜SPME等方面的研究进展,并对COFs材料在SPME领域的发展趋势进行了展望。

## 1 基于COFs材料的纤维SPME

### 1.1 环境样品的分析检测

环境污染已成为一个全球性的问题,各类污染物对人类健康和生态系统构成潜在风险,这一现状引起了研究人员的广泛关注。大多数环境污染物(如邻苯二甲酸酯(PAEs)、多卤联苯、多环芳烃(PAHs)等)具有致癌性和致畸性,会对人类健康造成很大威胁。然而,环境污染物的水平较低且样品基质复杂,因此开发高效的样品前处理方法至关重要。

Zhang等^[11]^制备了一种由喹啉桥接的COFs材料(N-QTTI-COF),将该材料固定在不锈钢丝上,制成SPME纤维(图1)。基于N-QTTI-COF与PAEs之间的*π-π*相互作用和强疏水作用,该方法可用于PAEs的固相微萃取。利用气相色谱-质谱(GC-MS)技术,该方法可实现PAEs的高灵敏检测,其中检出限(LOD)为0.17~1.70 ng/L,定量限(LOQ)为0.57~5.60 ng/L, PAEs在环境水样和工业废水中的加标回收率为90.0%~106.2%。为了快速制备COFs涂层纤维,Yu等^[12]^发展了一种*β*-酮烯胺修饰COFs材料的制备新策略,该策略可在1 h内制备出4种不同的COFs(TpTph-COF、TpPa-1-COF、TpBD-COF和TpTpb-COF)涂层纤维;随后,Yu等^[12]^将TpTph-COF涂层纤维作为SPME材料,并利用气相色谱-串联质谱(GC-MS/MS)技术实现了环境水中微量PAEs的快速萃取和高灵敏测定,所得到的LOD为0.02~0.08 ng/L,加标回收率为82.2%~117.5%,相对标准偏差(RSD)≤9.4%(*n*=6)。Yu等^[13]^利用四(4-氨基苯基)卟啉与4,4'-联苯二甲醛之间的席夫碱反应合成了一种卟啉基COFs材料,该材料可与PAHs之间产生*π-π*相互作用,将其涂覆在不锈钢上可用于PAHs的顶空固相微萃取(HS-SPME)。将HS-SPME技术与气相色谱-火焰离子检测器(GC-FID)结合,建立了水和土壤样品中PAHs的测定方法,结果表明,卟啉基COFs材料对PAHs的萃取效果较好,线性范围为1~150 ng/mL, LOD为0.25 ng/mL, LOQ为0.5 ng/mL。

**图 1:**
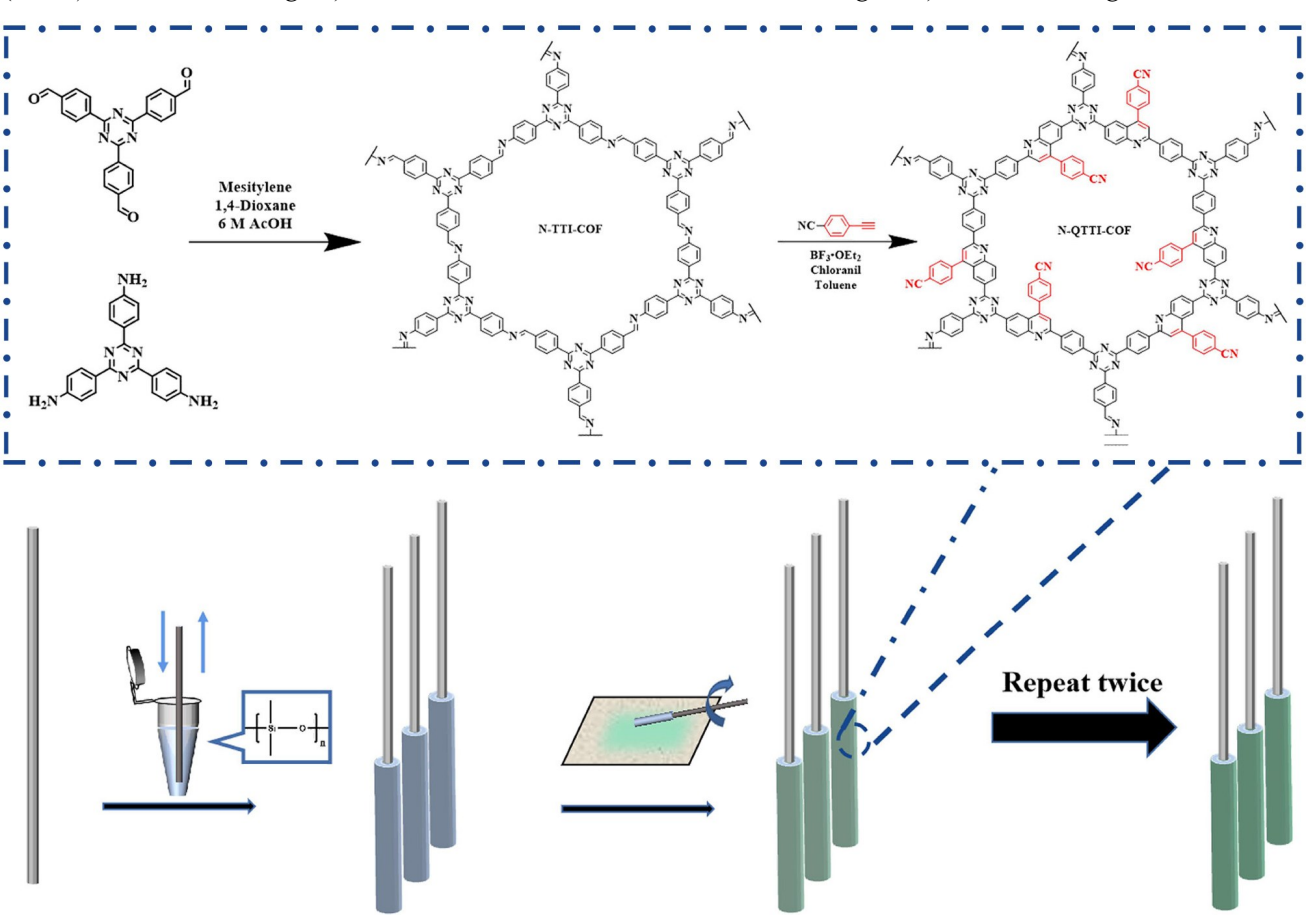
N-QTTI-COF的合成及SPME纤维的制备示意图^[11]^

由于采用物理涂覆方式制备的萃取纤维存在涂层黏接不牢固等缺点,因此需要探索使用交联剂固定或化学键合的方法来改进制备工艺。Li等^[14]^以(3-氨丙基)三乙氧基硅烷(APTES)为交联剂,将TpBD-COF修饰到不锈钢丝上,制备成SPME纤维,并基于该SPME纤维和GC-FID技术,建立了水样中7种微量PAHs的测定方法。实验结果表明,所制备的SPME纤维具有较好的萃取重现性,所获得的LOD为1.0~5.0 μg/L,回收率为87.59%~162.92%。杨梦奇等^[15]^先利用APTES对电化学腐蚀的不锈钢丝进行氨基化处理,然后利用化学键合的方式将TpPa-COF修饰到不锈钢纤维表面,制备了一种新型SPME纤维。TpPa-COF的孔径较大,能够提供充足的吸附位点和*π-π*相互作用,因此该纤维对PAHs展现了出较高的萃取能力。基于该SPME纤维和GC-FID技术,该研究实现了当地湖水、自来水和饮用水中7种PAHs的检测,所得到的回收率为95.8%~165.4%。大多数金属有机框架(MOFs)材料的化学稳定性和热稳定性相对较低,这限制了MOFs材料在SPME领域的应用。为了克服这一缺陷,Koonan等^[16]^将锌基MOF与三聚氰胺基COF结合,制备了一种Zn-MOF/COF杂化材料;利用物理涂覆法将该杂化材料固定在不锈钢丝上,制成SPME纤维,并应用于土壤样品中PAHs的萃取、富集和检测。结果表明,该SPME纤维的制备重复性和萃取重复性均较好,所获得的LOD和回收率分别为0.1~1 ng/g和91.1%~110.2%。此外,与商品化SPME纤维相比,该自制SPME纤维的成本更低,萃取效率更高。Su等^[17]^将氨基甲酰间苯三酚(Tp)和2,5-二氯-1,4-苯二胺作为原料,在氨基功能化的不锈钢丝上原位制备了氯功能化的COFs(CF-COF)涂层纤维。基于CF-COF与多氯联苯(PCBs)之间的强疏水相互作用、尺寸匹配效应、微孔效应和*π-π*相互作用,并利用GC-MS技术,该研究建立了环境地表水样中17种超痕量PCBs的分析检测方法。结果表明,CF-COF涂层纤维对17种PCBs的富集倍数(EFs)为699~4281,方法的线性范围和LOD分别为0.1~1000 ng/L和0.0015~0.0088 ng/L。将该方法应用于实际样品的分析检测,所得到的回收率为78.7%~124.0%。

由于空间作用的限制,二维COFs对非共面化合物的萃取效率较低。为解决这一问题,Lu等^[18]^将四(对氨基苯基)甲烷(TAM)和醛类试剂作为单体,利用席夫碱反应合成了4种三维COFs,并将其中一种由*β*-酮胺连接的Tp-TAM COF材料作为SPME涂层,结合HS-SPME-GC-MS技术,实现了河流水体和土壤样品中PCBs的检测。该SPME涂层与PCBs之间存在*π-π*共轭、疏水作用、卤素键和空间选择性等多种作用力,展现出了良好的萃取性能。结果表明,该方法具有较低的LOD(0.001~0.020 ng/L)和LOQ(0.004~0.066 ng/L),将该方法应用于河流水体和土壤样品中PCBs的检测,所得到的加标回收率分别为84.8%~117.2%和84.4%~114.7%。精确调控COFs的微结构是一个颇具吸引力的研究方向。在COFs的合成过程中,可以通过加入调节剂来优化比表面积、结晶度和结构尺寸。Zhou等^[19]^将2,5-二甲氧基苯甲醛(DB)作为调节剂,2,5-二甲氧基对苯二甲醛(DMTP)和1,3,5-三(4-氨基苯基)苯(TAPB)作为单体,在温和的条件下合成了具有高比表面积和强疏水性的TAPB-DMTP-DB COFs。将TAPB-DMTP-DB COFs制备成形貌均一、厚度均匀的SPME涂层,并与GC-MS技术结合,可用于环境水样中超痕量多溴联苯(PBBs)的精确检测。结果表明,所制备的TAPB-DMTP-DB COFs对PBBs展现出了较高的富集能力(EFs为4400~11360);并且,该方法的重复性较好,LOD低(0.04~0.28 ng/L),线性范围宽(0.25~5000 ng/L)。Song等^[20]^将1,3,5三(4-氨基苯基)苯(TPB)和DMTP作为反应物,采用室温合成法在不锈钢丝上原位合成了COFs涂层;随后,他们采用简单的浸渍法在COFs涂层上覆盖了超薄聚酰亚胺(PI)涂层,制备成SS-PI@TPB-DMTP SPME纤维(图2)。将该SPME纤维与气相色谱-负化学电离源-质谱(GC-NCI-MS)技术结合,建立了环境水样中6种多溴二苯醚(PBDEs)的分析检测方法。实验结果表明,该方法具有较宽的线性范围(0.05~100 ng/L)、较高的EFs(1470~3555)、较低的LOD(0.0083~0.0190 ng/L)和较好的回收率(79.2%~117.3%)。此外,该SPME纤维的制备过程简单且绿色环保,制备重复性和萃取重复性均较好,其在环境分析领域具有较大的应用潜力。

**图 2:**
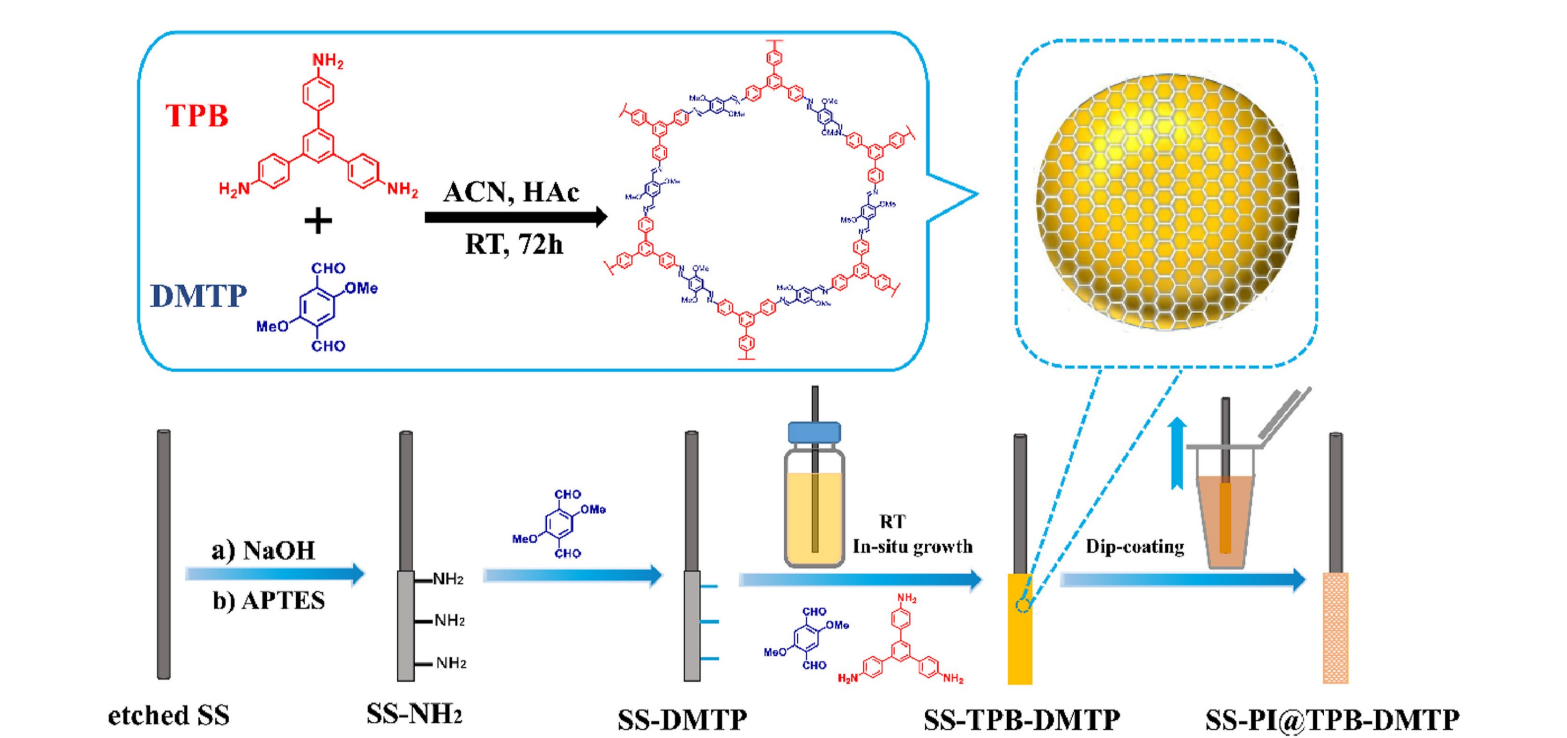
SS-PI@TPB-DMTP SPME纤维的制备流程图^[20]^

通过对COFs进行功能化修饰,可以精确地调控其萃取性能。Su等^[21]^利用光聚合法将聚(1-乙烯基-3-甲基甲基咪唑双(三氟甲基磺酰基)亚胺盐)(PIL)连接到乙烯基修饰的COFs上,合成了PIL-COF杂化材料。该杂化材料保持了COFs的结晶度和孔隙率,能够为目标分析物提供充足的吸附位点;同时,该杂化材料中的PIL可以与目标分析物之间产生多种相互作用力(包括范德华力、氢键和疏水相互作用),促进了对目标分析物的协同捕获。将PIL-COF用于水中PBDEs的萃取和检测,获得了较宽的线性范围(0.01~100 ng/L)、较低的LOD(0.0021~0.014 ng/L)和较高的回收率(78.6%~103.6%)。此外,该研究通过密度泛函理论(DFT)揭示了PIL-COF对PBDEs的捕获机制,为COFs杂化材料的设计提供了新的见解。上述研究结果表明,通过对COFs进行功能化修饰可以增强它们与目标分析物之间的相互作用,从而提高对分析物的萃取能力。

Guo等^[22]^使用原位合成和物理涂覆两种方法分别在不锈钢丝表面制备TpBD-COFs涂层纤维,随后将两种涂层纤维用于苯酚类化合物的萃取。结果表明,与通过物理涂覆法制备的涂层纤维和商品化聚丙烯酸酯涂层纤维相比,原位合成法制备的COFs涂层纤维的萃取性能更加优越,原因是在原位合成过程中没有使用任何黏接剂,COFs的多孔结构得到了良好的保留。将上述通过原位合成法制备的TpBD-COFs涂层纤维与GC-MS技术结合,用于水和土壤中苯酚类化合物的分析测定。结果表明,该TpBD-COFs涂层纤维对苯酚类化合物具有较高的EFs(11080~58762),苯酚类化合物的线性范围为2~10000 ng/L, LOD为0.39~0.72 ng/L;并且,TpBD-COFs涂层纤维的原位合成过程十分简便、环保,为设计出性能更优的SPME涂层提供了基础。Yang等^[23]^制备了由喹啉连接的超稳定二维COFs(CN-COF)涂层纤维,并将其用于环境水中14种有机氯农药(OCPs)的萃取和富集。与商品化SPME涂层纤维(PDM、CAR/PDMS、PDMS/DVB、DVB/CAR/PDMS)相比,CN-COF涂层纤维对14种OCPs的萃取效率更高(EFs为540~5065)。在最佳萃取条件下,CN-COF涂层纤维可连续使用70次,具有较好的稳定性。此外,基于CN-COF涂层纤维和HS-SPME-GC-MS/MS技术,该研究实现了14种OCPs的高灵敏检测(LOD为0.0010~13.54 ng/L)。

为了提高COFs对目标分析物的萃取选择性和效率,Song等^[24]^根据目标分析物的特性设计合成了新型功能化COFs材料(NH-CO-F9-COF)。将NH-CO-F9-COF作为SPME材料,并基于超高效液相色谱-串联质谱(UHPLC-MS/MS)技术,建立了一种简单、灵敏的全氟烷基和多氟烷基化合物(PFASs)分析方法,所获得的EFs为66~160, LOD为0.0035~0.18 ng/L。利用该方法对实际水样中的PFASs进行检测,所得到的回收率为77.1%~108%,相对标准偏差(RSD)≤11.4%。Gao等^[25]^利用Tp、苯二胺(Pa)和联苯胺(BD)构建了孔径可调的多组分COFs材料(MC-COFs),通过调节BD的比例可以控制MC-COFs的孔径大小。当孔径尺寸与分析物尺寸相匹配时,吸附效果最佳。与孔径较小的TpPa-COFs、孔径较大的TpBD-COFs和商业化涂层纤维(如PDMS/DVB/PA和PDMS)相比,TpPaBD_50_-MC-COFs(BD的加入比例为50%)对四溴双酚A(TBBPA)衍生物的选择吸附性更佳。将TpPaBD_50_-MC-COFs与恒流解析电质谱(CFDI-MS)联用,建立了TBBPA的分析方法,该方法具有低的LOD和LOQ(0.5~12 ng/L和1.6~40 ng/L)。与其他方法相比,该方法缩短了萃取和分析时间(7 min),可应用于实际水样中TBBPA及其衍生物的检测。Hu等^[26]^制备了一种比表面积高、热化学稳定性好和富电子杂原子丰富的COFs材料(DaTp-COF),并将其作为SPME涂层纤维,用于芳香酯类分析物的萃取。与商业化涂层纤维(PDMS和PDMS/DVB)相比,DaTp-COF涂层纤维对芳香酯类分析物的萃取效率更高。将DaTp-COF涂层纤维与GC-MS技术联用,建立化妆品塑料包装中多种芳香酯类化合物的测定方法,所获得的线性范围为0.002~1000 μg/L, LOD为0.00048~1.0 μg/L,回收率为80.7%~118%。

### 1.2 食品样品的分析检测

随着食品安全供应链的日益复杂化和各种新型食品污染物的不断涌现,食品安全问题愈发令人担忧。其中,食品中的农药残留已成为当前最为突出的问题之一。此外,部分环境污染物也会通过生态循环等途径进入到动植物体内,进而对人类健康造成威胁。因此,食品样品的分析检测备受关注。

氧化石墨烯(rGO)纳米片的堆积会导致其萃取性能显著降低,为了克服这一缺点,Yu等^[27]^采用超声自组装技术制备了COF/rGO复合材料,将其涂敷在不锈钢丝表面,制备成COF/rGO复合纤维,并用于PAHs的HS-SPME。基于GC-FID技术,研究建立了PAHs的分析测定方法,并获得了较宽的线性范围(0.5~250.0 ng/mL)和较低的LOD(0.09~0.59 ng/mL)。将该方法应用于蜂蜜中PAHs的含量测定,获得了满意的回收率(62.99%~128.8%)。研究中所制备的COF/rGO复合纤维有效阻止了rGO纳米片的堆积,提高了萃取效率,为复杂样品中痕量PAHs的分析提供了新的思路。Yu等^[28]^制备了一种新型MOF/COF_1_复合材料(图3),并将其制备成SPME纤维。基于该复合材料与PAHs之间的*π-π*相互作用、疏水相互作用和范德华力,该复合材料对PAHs表现出了较好的萃取效果。基于此,建立了5种PAHs的HS-SPME-GC-FID分析方法,并获得了较宽的线性范围(0.2~50.0 ng/mL)和较低的LOD(0.02~0.07 ng/mL)。将该方法应用于牛奶样品中PAHs的检测,获得了满意的回收率(85.43%~115.8%)。Xu等^[29]^在铁丝表面原位合成了一种新型球形MOF(NH_2_-MIL88),将COFs修饰到NH_2_-MIL88表面,制备成IW@NH_2_-MIL88-COF复合涂层纤维。该复合涂层纤维具有多孔结构,且其与PAHs之间存在疏水相互作用和强*π-π*堆积作用,因此该复合涂层纤维对PAHs展现出了良好的萃取性能。基于此,建立了苊、萘、菲、苊烯、芴5种PAHs的HS-SPME-GC-FID分析方法,该方法的线性范围为1~200 ng/mL, LOD为0.017~0.028 ng/mL。将该方法应用于牛奶样品中PAHs的检测,获得了满意的回收率(64.69%~113.97%)。该方法所制备的复合涂层纤维在有机污染物的分析和检测方面有很大的应用前景,并为构建多功能复合材料提供了新的思路。Zhao等^[30]^通过在二维MXene纳米片表面原位生长COF,制备了COF@Ti_3_C_2_T_X_复合材料。基于独特的结构特征和多种作用力(*π-π*相互作用、疏水相互作用和金属络合作用等),该复合材料可用于PAHs的HS-SPME。将该复合材料与GC-FID技术联用,建立了蜂蜜样品中PAHs的分析测定方法。实验结果表明,该方法具有较宽的线性范围(2.0~2000 ng/g)、较低的LOD(0.20~0.60 ng/g)和较高的回收率(73.2%~112%)。该研究成功解决了原位生长过程中涂层厚度的控制难题,并为复杂食品样品中PAHs的检测提供了一种新的思路。上述研究均通过将COFs与其他材料结合来提高杂化材料的萃取效率,并成功应用于实际样品的分析检测。

**图 3:**
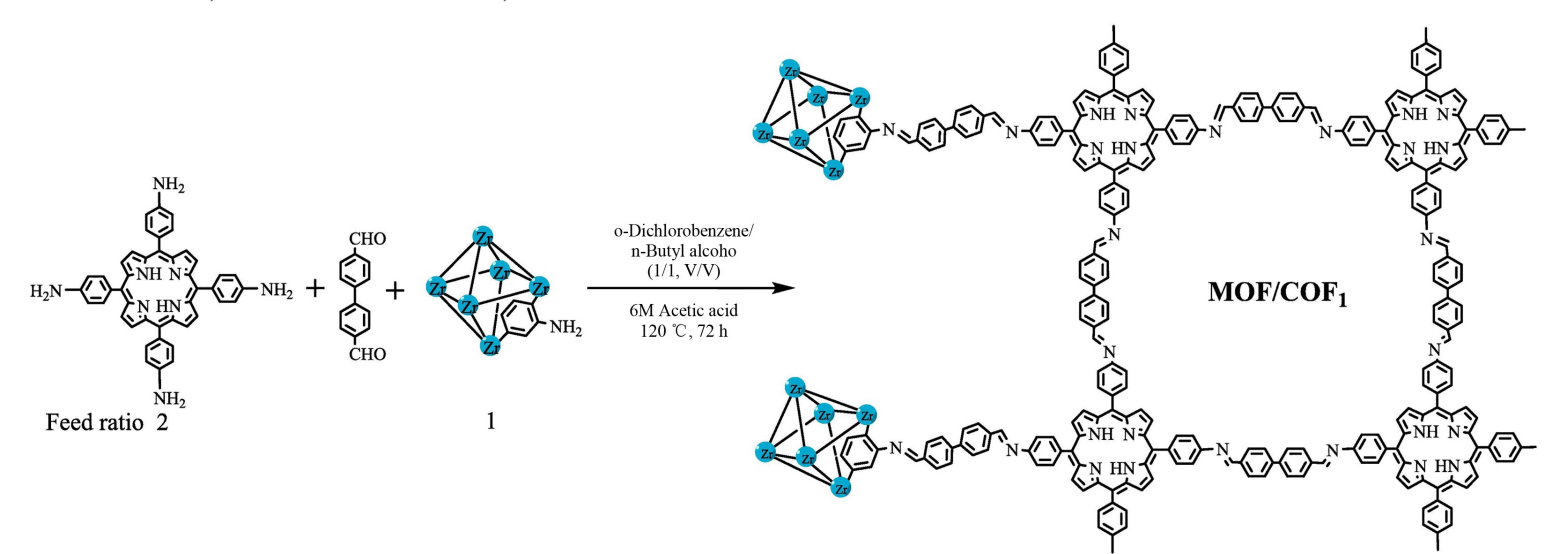
MOF/COF_1_复合材料的合成示意图^[28]^

鉴于国内外对进出口茶叶中农药残留限量要求的日益严格,开发出一种用于超高效捕获茶叶中拟除虫菊酯类杀虫剂(PYs)的SPME涂层具有重要意义。Yu等^[31]^在室温下通过原位快速合成法,在30 min内制备出了亚胺连接的COFs(TPB-DVA COF)涂层纤维。基于*π-π*相互作用、疏水相互作用和氢键等多重作用力,TPB-DVA COF涂层纤维对PYs展现出了良好的萃取效果(EFs为2700~13195)。基于此,建立了茶叶中5种PYs的SPME-GC-MS/MS分析方法,结果表明,该方法的线性范围为0.08~800 ng/L, LOD为0.02~0.20 ng/L,实际样品中的加标回收率为80.2%~119.8%。Li等^[32]^利用溶剂热反应合成了一种新型富氮亚胺COFs材料(N-COF),并通过物理涂覆法将N-COF固定在不锈钢丝表面,制备成N-COF SPME涂层纤维。N-COF中具有丰富的N原子和*π*电子,能够对OCPs产生很强的吸附作用。基于此,将N-COF SPME涂层纤维与气相色谱-电子捕获检测器(GC-ECD)联用,建立长叶莴苣、白菜、大白菜、苹果、梨和桃样品中OCPs的分析测定方法,所获得的线性范围为0.1~100.0 ng/g, LOD为0.03~0.3 ng/g,回收率为73.7%~111.6%。实验结果证实,作为一种高效的吸附剂,N-COF在食品分析领域具有广阔的应用前景。Xin等^[33]^开发了一种富含N原子的COFs材料(Tp-A_ZO_ COF),并将其用作SPME涂层。得益于卤素键和疏水相互作用的协同效应,Tp-A_ZO_ COF对OCPs展现出了优异的萃取性能。基于此,研究建立了痕量OCPs的SPME-GC-MS/MS分析方法,该方法具有较宽的线性范围(0.1~1000 ng/L)、较低的LOD(0.002~0.08 ng/L)和较高的准确度。将该方法应用于牛奶、绿茶、自来水和井水4种实际样品中痕量OCPs的检测,获得了满意的回收率(83.4%~101.6%)。Wu等^[34]^通过在Ti_3_C_2_T_X_ MXene纳米片上组装*β*-酮烯胺连接的COF,制备了一种具有异质结构的MXene/COF(Ti_3_C_2_T_X_/TAPT-TFP COF)复合材料,随后将该复合材料逐层涂敷到硅羟基功能化的不锈钢基体上,制备成SPME纤维。该SPME纤维集成了MXene和COF的分层多孔结构及高比表面积,结合卤素键和范德华力等作用力,该SPME纤维可用于OCPs的萃取。基于该SPME纤维和GC-MS技术,建立了不同水果和蔬菜中OCPs残留的分析方法。结果表明,该方法具有较低的LOD(0.036~0.126 ng/g)和LOQ(0.12~0.42 ng/g)、较宽的线性范围(0.12~20.0 ng/g)以及较高的回收率(92.0%~104.2%)。

Tabibi等^[35]^以三聚氰胺、4,4'-乙二胺和3,4,9,10-苝四羧酸二酐为原料,在密封管内合成了新型多孔COF材料(PTA/TAPTT COF),并将其作为SPME纤维。基于该SPME纤维和GC-MS技术,研究建立了农药氟虫灵和毒死蜱的萃取和分析方法。实验结果表明,所制备的SPME纤维具有良好的热稳定性、大的比表面积以及良好的结晶度,对氟虫灵和毒死蜱展现出了较高的萃取能力,并获得了较宽的线性范围(氟虫灵:0.45~20 μg/L,毒死蜱:0.50~25 μg/L)和较低的LOD(氟虫灵:0.13 μg/L,毒死蜱:0.15 μg/L)。将该方法用于胡萝卜、黄瓜和葡萄等实际样品中氟虫灵和毒死蜱的萃取和测定,获得了较高的回收率(87%~110%)。Chen等^[36]^通过一步电聚合法在不锈钢纤维表面原位合成了高导电性的卟啉基COF(POR-COF)涂层。POR-COF由于具有优异的导电性、良好的结晶度和丰富的*π*电子,其对6种PAEs具有较高的萃取能力。将POR-COF与电增强SPME(EE-SPME)、GC-MS/MS技术结合,建立了实际样品中PAEs的分析方法,所获得的线性范围为0.2~2000 ng/L, LOD为0.05~2.0 ng/L,回收率为81.4%~118.4%。Wang等^[37]^合成了一种由哌嗪连接、铜离子掺杂的酞菁COF材料(CuPc-MCOF),基于CuPc-MCOF与氯酚类化合物(CPs)之间的*π-π*相互作用、氢键和金属配位等多重作用力,其可用于CPs的EE-SPME。基于此,研究建立了海水和海产品中痕量CPs的GC-MS/MS分析方法,该方法具有较宽的线性范围(2.0~1000.0 ng/L)和较低的LOD(2.0~1000.0 ng/L),在实际样品检测中,CPs的回收率为84.1%~116.5%, RSD<8.4%。Song等^[38]^通过化学还原法制备了由烯基连接的氨基功能化COF(NH_2_-COF),并将其作为SPME涂层,建立了PFASs的UHPLC-MS/MS分析方法。结果表明,该方法具有较宽的线性范围(1.0~1000 ng/g)、较低的LOD(0.001~0.006ng/g)和LOQ(0.005~0.02 ng/g)。

将该方法应用于6种海鱼样品中PFASs的分析检测,获得了满意的回收率(88.6%~111%)。Han等^[39]^合成了一种吡啶功能化的COF材料(Py-COF),该COF材料具有良好的结晶度、高的比表面积和丰富的活性吸附位点,可用于全氟聚醚羧酸(PFECAs)的固相微萃取。将Py-COF与HPLC-MS/MS技术结合,建立了食品中痕量PFECAs的分析方法,所获得的线性范围为0.005~7.5 ng/g, LOD为0.001~0.004 ng/g, LOQ为0.003~0.012 ng/g。将该方法用于马铃薯、生菜、黄瓜、梨、橘子、香蕉、鸡肉、牛肉、带鱼等食品样品中痕量PFECAs的检测,获得了较高的回收率(82.5%~112%)。Li等^[40]^合成了三维羟基功能化COF材料(3D-OH-COF),该COF材料中的吸电子原子(N、O)和羟基能够提供丰富的氢键作用。将3D-OH-COF与高效液相色谱-二极管阵列检测器(HPLC-DAD)结合,建立了苹果和大米样品中醋氨脒、甲氧苄啶和阿米咯的测定方法。结果表明,该方法具有较宽的线性范围(5.0~1000 ng/g)、较低的LOD(0.86~1.38 ng/g)和较好的回收率(79.3%~106.8%),在复杂食品样品中醋氨脒、甲氧苄啶和阿米咯的测定方面具有良好的应用前景。Mo等^[41]^以均苯三甲醛和对苯二胺为单体合成了LZU1-COF材料,随后通过物理涂覆法将其固定到不锈钢丝上,并使用透析膜进行保护,最后将该材料用于雌二醇(E2)的固相微萃取,具体如图4所示。COF-LZU1具有丰富的苯环和亚胺基团,可以提供*π-π*和疏水相互作用,其对E2具有很强的萃取能力。将COF-LZU1与GC-FID结合,建立了牛奶中E2的分析检测方法。该方法具有较宽的线性范围(5~800 μg/L)、较低的LOD(0.8 μg/L)和LOQ(2.5 μg/L)及较高的加标回收率(77.27%~108.26%)。在该研究中,保护膜有效防止了萃取涂层的脱落,增强了涂层纤维的稳定性。Zhou等^[42]^首次将4-甲酰基苯甲酸(FBA)作为调节剂,制备了一系列具有较高结晶度的羧基功能化COFs(FBA-COF-xs),根据表征结果选择萃取效果最好的FBA-COF-30%(FBA的添加比例为30%)作为萃取纤维。基于*π-π*相互作用、尺寸排斥效应和强极性相互作用,FBA-COF-30%萃取纤维对硝基苯化合物(NBCs)表现出了较好的富集效果。基于SPME-GC-MS/MS技术,研究建立了环境水样、牛奶样品和水果样品中7种NBCs的分析测定方法,并获得了较宽的线性范围(0.50~5000 ng/L)和较低的LOD(0.15~3.0 ng/L)。将该方法应用于实际样品的测定,均获得了令人满意的回收率,其中7种NBCs在环境水样中的回收率为80.0%~116.8%。研究结果表明,该方法有望应用于各种基质中NBCs的精确检测。Pang等^[43]^将2,6二氨基蒽醌(DQ)和1,3,5-三甲酰基间苯三酚(TP)作为单体,合成了具有导电性的COF材料,通过易溶液相法将该COF材料原位黏接在不锈钢丝上,用作EE-SPME涂层纤维。将该涂层纤维与GC-FID联用,用于内分泌干扰物双酚A(BPA)的萃取和检测,所获得的线性范围为0.05~10 μg/mL, LOD为0.003 μg/L。此外,将该方法应用于食品包装(矿泉水瓶、牛奶盒和奶茶杯)中BPA的测定,得到的回收率为88.6%~118.0%。

**图 4:**
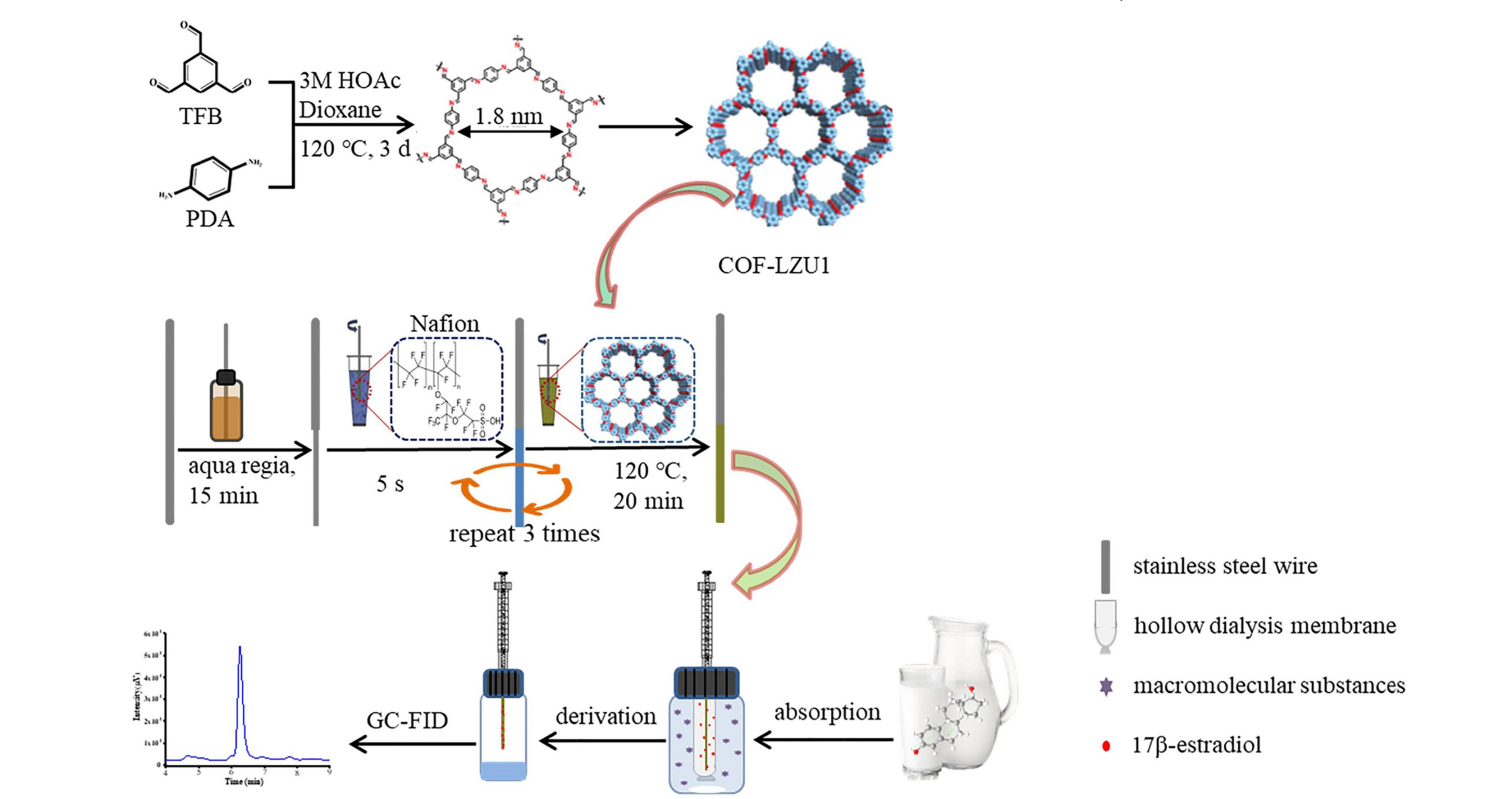
COF-LZU1涂层纤维的制备及牛奶样品中E2的检测示意图^[41]^

### 1.3 生物样品的分析检测

生物样品基质复杂,在分析检测之前,需对样品进行前处理。近年来,将COFs作为微萃取涂层的方法在生物领域逐渐得到了应用。血液样品中的药物分析检测会受到蛋白质等生物大分子的干扰,为了解决这一问题,Quan等^[44]^以TPB和2,5-二羟基对苯二甲酸为原料合成了一种富含羟基的COF材料(DES-1-COF),并将其用作SPME涂层纤维。DES-1-COF具有合适的孔径和丰富的官能团,其对木犀草素和槲皮素具有良好的吸附能力,最大吸附量分别为72.66 mg/g和42.38 mg/g。此外,DES-1-COF具有大尺寸排斥特性,其对7种选定的蛋白质表现出了良好的排斥效果(基体效应减小了93%)。基于SPME-HPLC-MS技术,研究建立了木犀草素、槲皮素及其代谢物的分析测定方法,所获得的线性范围为0.1~150 μg/mL, LOD为10~50 ng/mL, LOQ为25~100 ng/mL。实验结果表明,该SPME涂层纤维可有效萃取小鼠血液样品中的木犀草素、槲皮素及其代谢物;并且,与传统的蛋白质沉淀方法相比,该方法可以更有效地去除样品基质。Li等^[45]^合成了一种磁性COF纳米杂化体(NiFe_2_O_4_@COF),其比表面积为169.7 m^2^/g,利用聚二甲基硅氧烷和硅橡胶固化剂将其均匀地固定到石英丝上,制备成SPME纤维。研究以EFs为评价指标,分别考察了该SPME纤维对5种PYs(甲氰菊酯、联苯菊酯、氯菊酯、氰戊酸酯和溴氰菊酯)、三氯生(TCS)和甲基三氯生(MTCS)的萃取选择性。结果显示,TCS和MTCS的EFs为279~334,而5种PYs的EFs仅为76~147;进一步建立了TCS和MTCS的SPME-GC-ECD分析测定方法,所获得的线性范围为0.1~1000 μg/L, LOD和LOQ分别为1~7 ng/L和3.3~23 ng/L。将该方法应用于成年人和儿童尿液中TCS和MTCS的检测,获得了满意的回收率(81.9%~109.1%)。此外,该SPME纤维的萃取效率接近100%,远高于其他3种商业化SPME纤维(PDMS、PDMS/DVB和PDMS/DVB/CAR),且该SPME纤维具有优异的稳定性,可循环使用150次以上。该研究为复杂生物样品中的痕量环境污染物监测提供了一种方便、灵敏、高效、绿色的预处理方法,具有重要的应用前景。

阿米替林(AT)、多塞平(DOX)、去甲替林(NT)等三环类抗抑郁药(TCAs)是临床上治疗抑郁症的首选药物。为了提升治疗效果并明确患者死亡原因,监测生物体液及组织中的TCAs水平具有重要意义,因此开发一种生物样品中快速、灵敏的TCAs实时监测方法至关重要。Yuan等^[46]^采用原位分步合成法,将TPB和2,5-二乙烯基苯甲醛(DVA)依次修饰到聚多巴胺表面,制备了一种新型COF基SPME探针(TPB-DVA COF SPME)。该SPME探针具有较高的比表面积(1244 m^2^/g)、均一的孔径(3.23 nm)和较好的稳定性,可用于TCAs的高效富集。将TPB-DVA-COF SPME探针与质谱技术相结合,该研究首次实现了小鼠血清及组织中TCAs的快速、灵敏测定。结果表明,所制备的SPME探针具有较好的重复使用性和较高的EFs(39~218),同时所建方法的LOD较低(0.1~0.5 μg/L),能够作为药物监测的有力工具;利用该方法对小鼠血清及组织等实际样品中的TCAs进行测定,所获得的回收率为88.8%~114.7%, RSD为1.2%~3.8%。

## 2 基于COFs材料的管内SPME

管内SPME是SPME中的常见形式,其不仅能弥补纤维SPME的不足,还具有高稳定性、高灵敏度等特点,可与分析仪器实现在线联用。管内SPME的微萃取管主要分为几种类型:开管型、整体柱型、颗粒填充型以及纤维填充型。

目前,COF整体柱型微萃取管在样品制备领域具有良好的应用潜力,其主要吸附机制为疏水相互作用和*π-π*相互作用。Wang等^[47]^将TAPB-TPA COF微球包埋在脲醛树脂(UF)中,制备了一种新型COF基整体柱。基于该COF基整体柱和高效液相色谱-紫外(HPLC-UV)技术,研究建立了食用油中微量合成酚类抗氧化剂(SPAs)的在线分析方法。通过对萃取溶液和洗脱液中乙腈的体积比、洗脱液收集时间和萃取溶液pH等条件进行优化,获得了最佳萃取条件。实验结果表明,该COF基整体柱对SPAs具有较好的萃取效果;所建方法具有低的LOD(0.2~1.2 ng/mL),且在实际样品检测中获得了较高的回收率(78.1%~98.4%)和较低的RSD(<7.2%)。为了提高碳纤维对有机污染物的萃取效率,Sun等^[48]^首先在碳纤维上原位生长TiO_2_纳米棒阵列(TiO_2_ NARs),制得TiO_2_ NARs-CFs;随后,以TPB和2,5-二乙烯基对苯二甲醛为反应单体,利用席夫碱反应在TiO_2_ NARs-CFs上原位制备COF纳米球(COF nanosphere),得到COF nanosphere-TiO_2_ NARs-CFs,具体制备过程如图5所示。然后将COF nanosphere-TiO_2_ NARs-CFs填装至内径为0.75 μm的聚醚醚酮管中得到固相微萃取管,并将其用于4类常见有机污染物(PAHs、雌激素、双酚类、塑化剂)的管内固相微萃取。实验分别考察了COF nanosphere-TiO_2_ NARs-CFs对4种有机污染物的萃取性能,随后选择萃取效果最好(EFs高达3745)的双酚类化合物进行后续分析检测。通过六通阀将该固相微萃取管与HPLC进行在线联用,建立双酚类化合物的分析测定方法。结果表明,该方法的LOD为0.001 μg/L,线性范围为0.017~15.0 μg/L,在实际水样分析中,方法的RSD为0.2%~15.2%。虽然离线模式的SPME技术能够减少溶液的消耗,但它仍然依赖于人工处理,因此颇为不便。上述两项研究表明,通过构建在线SPME系统,可以实现萃取富集与分析的一体化操作。

**图 5:**
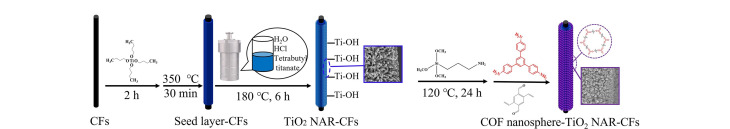
COF纳米球-TiO_2_ NAR-CFs的制备示意图^[48]^

## 3 基于COFs材料的膜SPME

膜SPME技术主要利用具有高比表面积的聚合物薄膜作为萃取材料,该技术对于水质中微量污染物的富集和检测至关重要,是评价水质的关键手段。Lv等^[49]^通过在胺化聚丙烯腈(PAN)纳米纤维上原位生长TpPa-COF,制备了一种新型纳米纤维膜(PAN-SiO_2_@TpPa COF)。该纳米纤维膜具有丰富的官能团(氨基、羟基和芳香基)、良好的热稳定性和化学稳定性,对PCBs展现出了较好的萃取性能(EFs为2714~3949),且可重复使用150次以上。基于该纳米纤维膜和GC-ECD技术,建立天然水体(河流、湖泊和海水)中痕量PCBs的分析测定方法。结果表明,PCBs的线性相关系数(*R*^2^)≥0.99, LOD为0.1~5 ng/L;此外,在实际水样检测中,该纳米纤维膜能够有效降低河流、湖泊和海水的基质效应。最后,本文列举了COFs材料在纤维SPME、管内SPME以及膜SPME等样品前处理技术中的应用,详见附表1(https://www.chrom-China.com)。

## 4 总结与展望

本文总结了近三年COFs在SPME领域的研究进展,介绍了COFs及其功能化或杂化材料在纤维SPME、管内SPME及膜SPME等样品前处理技术中的应用。将SPME技术与GC-MS、HPLC-MS等检测技术联用,可实现环境水、食品、土壤等样品中多种类型有机污染物(包括PAEs、PAHs、PFASs、PYs等)的萃取、富集和检测。未来COFs在SPME领域的发展趋势主要集中于以下几个方面:(1)开发简便、快速、绿色的COFs萃取涂层合成方法;(2)开发更多高性能的功能化COFs材料,以提升目标分析物的萃取选择性和萃取效率;(3)借助氧化石墨烯、MOF和金属氧化物等材料的优势,设计制备COFs杂化材料,拓展COFs在SPME领域的应用范围;(4)通过调控COFs的微观结构和化学组成(如孔径、比表面积、电荷密度、吸附位点、官能团等),发展快速、高效的SPME方法;(5)发展高稳定性的COFs材料,提高使用寿命和分析准确性;(6)调控COFs材料的孔径,使其能够在高效吸附小分子分析物的同时有效排阻基体中的大分子,降低基体效应,以用于生物样品的检测。(7)开发出更多可应用于管内SPME的COFs材料以及在线萃取和在线分析方法,以顺应分析化学发展的趋势;(8)将COFs基SPME材料拓展至更广泛的应用领域,特别是生命分析领域。
